# Exploring the mechanisms of desert plant adaptation to arid climates: a multi-omics analysis of dehydration and rehydration responses in *Syntrichia caninervis*

**DOI:** 10.1007/s44154-025-00241-w

**Published:** 2025-11-04

**Authors:** Qilin Yang, Huan Zhang, Fangliu Yin, Haron Salih, RuiRui Yang, Bei Gao, Xiaoshuang Li, Daoyuan Zhang

**Affiliations:** 1 State Key Laboratory of Ecological Safety and Sustainable Development in Arid Lands, Xinjiang Institute of Ecology and Geography, Chinese Academy of Sciences, Urumqi, 830011 China; 2 Xinjiang Key Lab of Conservation and Utilization of Plant Gene Resources, Xinjiang Institute of Ecology and Geography, Chinese Academy of Sciences, Urumqi, 830011 China; 3 University of Chinese Academy of Sciences, Beijing, 100049 China

**Keywords:** Desert moss, Desiccation tolerance, Extremophile adaptation, Extreme environment, Multi-omics integration

## Abstract

Desert plants have evolved remarkable adaptations to survive in arid environments, where water scarcity and extreme temperatures pose significant challenges to life. The desert moss *Syntrichia caninervis* stands out as an exemplary model of extreme desiccation tolerance (DT), offering invaluable insights into plant adaptation to water deficit. This study presents a comprehensive multi-omics analysis of *S. caninervis* during controlled dehydration and rehydration process, integrating transcriptomic, proteomic, and metabolomic data to elucidate the molecular mechanisms underlying its remarkable resilience. Our findings reveal a sophisticated, multilayered response characterized by extensive transcriptional reprogramming (3,153 differentially expressed genes), dynamic proteome remodeling (873 differentially expressed proteins), and strategic metabolic reconfiguration (185 differentially abundant metabolites). Key adaptations include the coordinated downregulation of photosynthetic processes, upregulation of stress-responsive genes and proteins, accumulation of protective metabolites, and enhancement of antioxidant systems. Notably, we observed significant temporal asynchrony between transcript and protein levels, underscoring the complexity of post-transcriptional regulation in stress responses. The core mechanisms of *S. caninervis* DT comprises cellular protection and metabolic dormancy during dehydration, followed by efficient repair and recovery processes upon rehydration. These findings not only advance our understanding of plant evolution and adaptation to extreme environments but also identify potential targets for enhancing drought tolerance in crops and exploring plant survival under extreme environment. By deciphering the molecular basis of extreme DT, this research opens new avenues for addressing agricultural challenges in water-limited environments and expands our knowledge of plant life's adaptability to harsh terrestrial.

**Supplementary Information:**

The online version contains supplementary material available at 10.1007/s44154-025-00241-w.

## Introduction

Earth's arid zones, covering approximately 41% of the global land surface, represent some of the most extreme environments on our planet and are at the forefront of climate change impacts (Pravalie et al. 2016; Huang et al. 2017). These regions, characterized by extreme temperature fluctuations, intense solar radiation, and severe water scarcity, serve as natural laboratories for studying the limits of life and plant adaptations to environmental stress. As climate change accelerates, understanding the mechanisms of extreme stress tolerance in desert-adapted organisms becomes increasingly crucial, not only for ecological insights but also for addressing future challenges in agriculture, conservation, and even space exploration (Mohanta et al. 2024). The study of extremophiles in these harsh environments provides a window into the remarkable adaptability of life and offers valuable lessons for surviving in a changing world.

Biological soil crusts (BSCs) cover 12% of Earth's terrestrial surface, playing a crucial role in arid ecosystems (Rodriguez-Caballero et al. 2018). BSCs contribute approximately 7% of terrestrial net primary production and about 50% of biological nitrogen fixation (Elbert et al. 2012). In arid regions, biocrusts dominate, providing soil stabilization, water retention, and nutrient cycling. The deser tmoss *Syntrichia caninervis*, dominant species of BSC abundant in the Gurbantunggut Desert of China, exemplifies extreme stress adaptation in these environments (Stark et al. 2005; Zhang et al. 2005; Zhang et al. 2006; Pan et al. 2016; Zhang et al. 2020; Coe. et al. 2021; Yang et al. 2023a, b; Li et al. 2024). This remarkable moss possesses an extraordinary capacity for desiccation tolerance (DT) (Oliver, et al. 2020; Farrant et al. 2021; Marks et al. 2024), surviving the loss of over 95% of its cellular water content and recovering full physiological function within seconds of rehydration (Li et al. 2024). *S. caninervis* has shown remarkable resilience to a variety of extreme environmental stresses (ultra-low temperatures, and high levels of gamma radiation) (Li et al. 2024; Pan and Lin. 2024). Moreover, *S. caninervis* has shown the ability to survive and maintain vitality under simulated Mars conditions (Li et al. 2024; Pan and Lin. 2024). Such extreme resilience makes *S. caninervis* an ideal model species for studying the limits of plant survival and the molecular mechanisms underlying stress adaptation. This concentration in an area of extreme aridity not only underscores the species'ecological importance but also positions it as a critical subject for understanding plant responses to global aridification trends. As climate change expands arid zones worldwide, the adaptive strategies of *S. caninervis* could provide crucial insights for predicting and mitigating the impacts on both natural ecosystems and agricultural systems.

Despite significant advances in our understanding of desiccation tolerance, critical gaps remain in our knowledge of the integrated cellular responses that enable survival under extreme water stress. Previous research on *S. caninervis* and other desiccation-tolerant plants has largely focused on individual molecular levels (Silva et al. 2021; Gao et al. 2024; Yang et al. 2023a, 2023b). This fragmented approach has limited our ability to comprehend the complex interplay between gene expression, protein abundance, and metabolite profiles during rapid dehydration and rehydration cycles. The lack of a comprehensive, systems-level analysis has left many questions unanswered: How do transcriptional changes correlate with protein abundance and metabolite production? What are the key regulatory hubs coordinating the desiccation response? How does *S. caninervis* achieve such rapid physiological recovery upon rehydration? Addressing these questions requires a novel, integrated approach that can capture the dynamic and multifaceted nature of extreme stress tolerance.

This study presents a multi-omics analysis of *S. caninervis* during controlled dehydration and rehydration, integrating transcriptomic, proteomic, and metabolomic data to elucidate the molecular basis of its remarkable resilience. By data integration methods, we aim to uncover the most critical pathways, genes, and strategies employed by *S. caninervis* in achieving extreme DT. This integrated approach has the potential to reveal novel insights into the coordinated cellular responses that enable survival under extreme water stress, potentially identifying key regulatory hubs and previously unrecognized protective mechanisms. Such findings could significantly deepen our theoretical understanding of plant DT and expand the practical applications of these adaptive strategies. The knowledge gained from this study may inform the development of innovative approaches to enhance crop resilience in water-limited environments, guide conservation efforts in the face of climate change, and even contribute to the design of bio-inspired solutions for space exploration and extraterrestrial colonization.

## Results

### Natural habitat of *S. caninervis* and physiological changes during dehydration-rehydration treatment

**Fig. 1:**
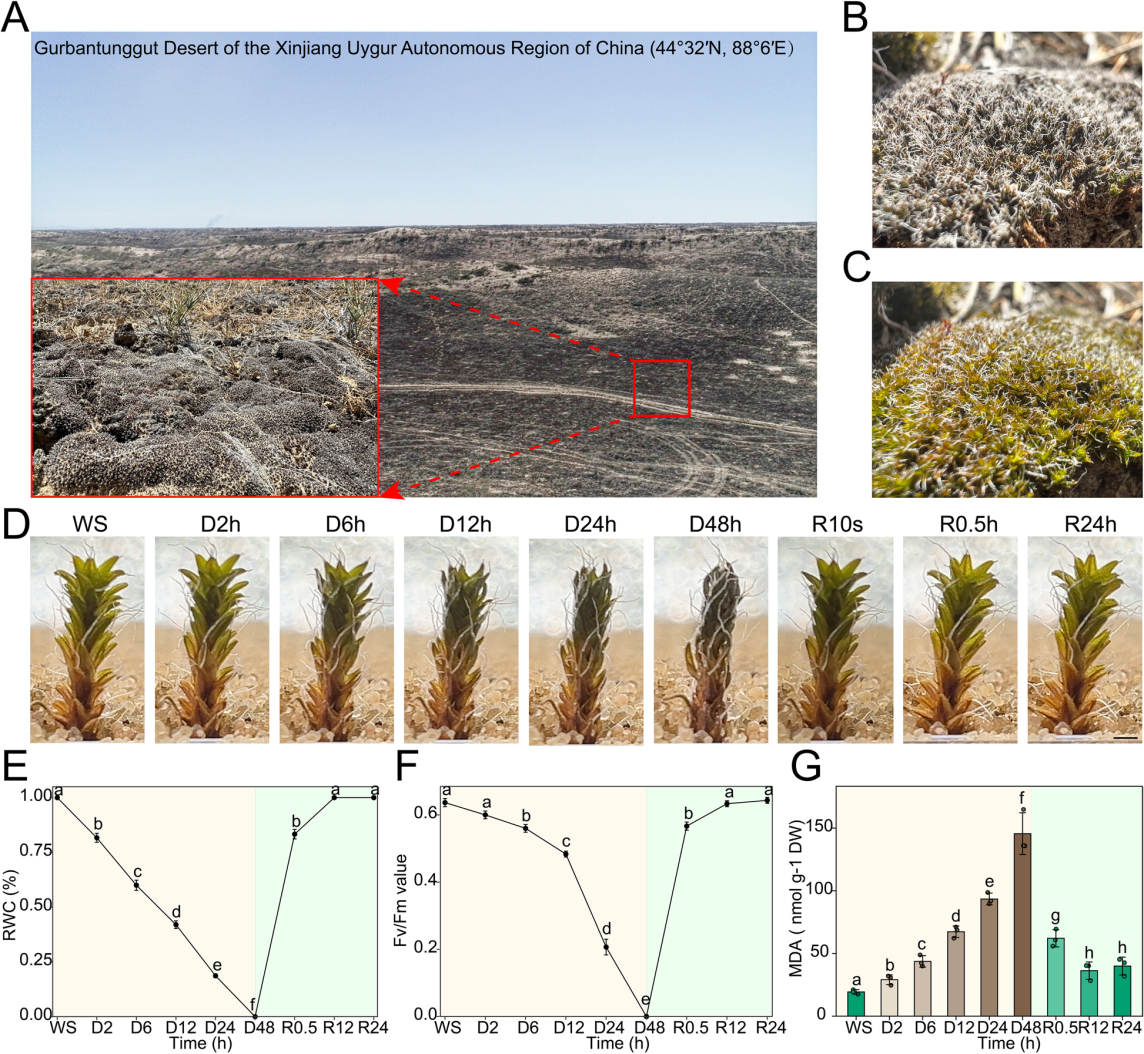
Habitat characteristics and physiological responses of *S. caninervis* during dehydration-rehydration (D-R) process. **A**. Representative landscape of the Gurbantunggut Desert (44°32′30"N, 88°6′42"E), Xinjiang, China, the natural habitat of *S. caninervis.* **B**. Desiccated *S. caninervis* crust. **C**. Hydrated *S. caninervis* crust. **D**. Phenotypic changes in individual *S. caninervis* plants during dehydration. **E**. Relative water content. **F**. Optimal photochemical efficiency of photosystem II value. **G**. MDA content. Dry *S. caninervis* samples were fully hydrated with ultrapure water for 24 h and air-dried in the laboratory (~ 30% relative humidity, 20–22 ℃) for dehydration treatment. Completely dry mosses were then watered to saturation for rehydration. Data are presented as means ± SEM, three biological replicates, *n* = 100 individual plants. Scale bars: 1 mm. Different lowercase letters indicate significant differences among treatments based on ANOVA followed by the Least Significant Difference test at *p* < 0.05

*S. caninervis* is widely distributed in the Gurbantunggut Desert in Xinjiang, China, which is characterized by large temperature fluctuations between day and night, as well as between seasons, and dry air conditions (Fig. 1A). In this harsh environment, *S. caninervis* remains in a completely dehydrated state for extended periods (Fig. 1B). However, upon rehydration, the moss quickly expands and recovers (Fig. 1C, D). To investigate the dehydration-rehydration (D-R) process of *S. caninervis*, a slow drying experiment was conducted. The results showed that the relative water content (RWC) (Fig. 1E) and optimal photochemical efficiency of photosystem II (*Fv/Fm*) (Fig. 1F) decreased with increasing dehydration time, approaching zero as the plant neared complete desiccation. Conversely, the malondialdehyde (MDA) content increased during dehydration, reaching a Maximum value of 145.52 ± 16.66 nmol g^−1^ DW at complete drying. Remarkably, after just 0.5 h of rehydration, the RWC and *Fv/Fm* values of *S. caninervis* recovered to 83% and 90% (0.57 ± 0.02), respectively. After 12 h of rehydration, both RWC and *Fv/Fm* had fully recovered, accompanied by a decrease in MDA content to 39.95 ± 7.12 nmol g^−1^ DW (Fig. 1G). The results highlight that the adaptation of this moss species to dry desert conditions comes from tolerance to dehydration and repair and recovery after rehydration.

### Transcriptome profiles of the dehydration and rehydration

**Fig. 2:**
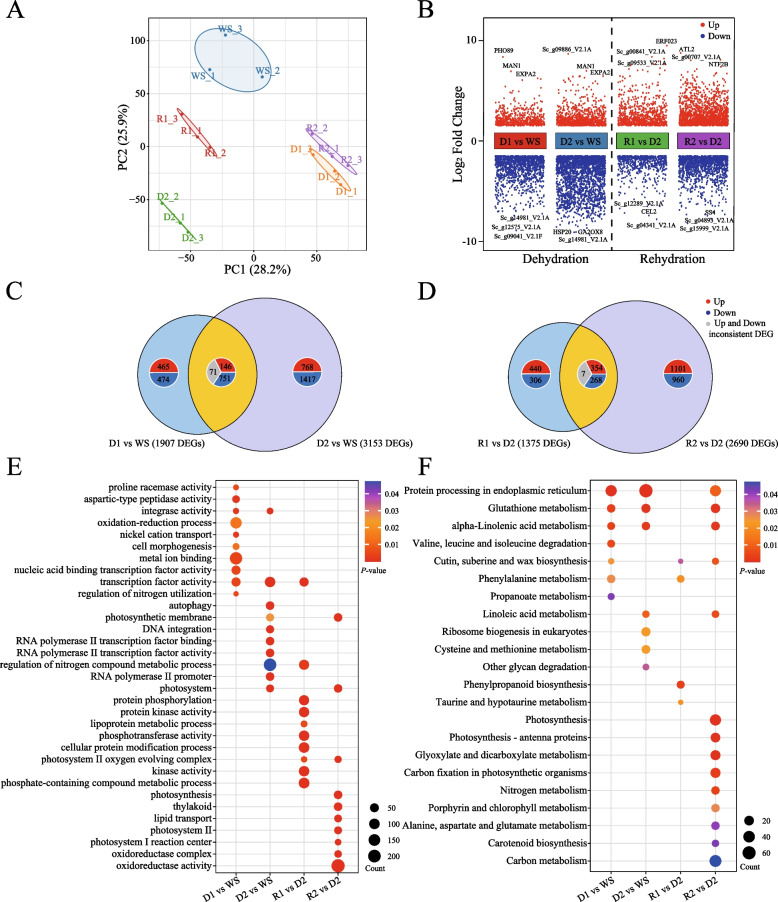
Transcriptome analysis of *S. caninervis* during D-R process. **A**. Principal Component Analysis (PCA) of RNA-Seq data. **B**. Analysis of the number of DEGs. **C**, **D**. Venn diagrams showing the number of DEGs. **E**. GO analysis of DEGs. Color intensity represents -log_10_(*p*-value) of enrichment. **F**. KEGG analysis of DEGs. Dot size indicates the number of DEGs in each pathway, while color represents the adjusted *p*-value

The de novo transcriptome assembly of *S. caninervis* was conducted to generate a reference for high-throughput gene expression analysis and protein identification. The transcriptome encompasses a total of 12,548 transcripts (Supplementary Material 1: Table S2). To validate the RNA-Seq data, real-time quantitative reverse transcription PCR (qRT-PCR) analysis was performed on ten genes involved in the D-R process. The gene expression levels determined by qRT-PCR showed a strong correlation with those obtained from RNA-Seq (Fig. S1). Principal component analysis (PCA) of the RNA-Seq data revealed differences in the overall gene expression profiles among various samples. Samples D1, D2, and R1, R2 were distinctly separated from WS, based on the first principal component (PC1), which accounts for 28.2% of the variance, and the second principal component (PC2), accounting for 25.9% of the variance. The clustering of WS and R1, R2 suggests that, at least at the transcriptional level, the plants recovered from desiccation and resumed most physiological processes within 24 h of rehydration; however, there were still some differences between WS and R2 (Fig. 2A). During dehydration, a significant difference in gene expression was observed, with 1907 differentially expressed genes (DEGs) in D1 compared to WS and 3153 DEGs in D2 versus WS, with 968 genes commonly showing differential expression. The rehydration process also exhibited notable changes, with 1375 DEGs detected in R1 relative to D2 and 2690 DEGs in R2 compared to D2, where 629 genes overlapped. These findings indicate that complete dehydration induced the most substantial alterations in gene expression (Fig. 2B, C and D). The application of machine learning-based analysis (GENIE3) to transcription factors-genes interactions revealed a complex regulatory network underlying gene expression during the D-R process in *S. caninervis* (Supplementary Material 1: Fig. S2). This network analysis identified several key transcription factor families playing pivotal roles in modulating D-R process genes. Notably, the network highlighted the prominence of AP2/ERF domain-containing factors, WRKY family transcription factors (particularly WRKY12), B3 domain-containing factors, bHLH (basic helix-loop-helix) transcription factors, and members of the HSF (Heat Shock Factor) family. Additional significant regulators included ARF (Auxin Response Factors), LFY (LEAFY), and C2H2 zinc finger proteins. The network structure revealed extensive branching and interconnections among these transcription factors and their target genes, indicating a high degree of regulatory complexity (Supplementary Material 1: Table S3).

During the D-R process, a notable upregulation of GO terms related to transcription factor activity, particularly RNA polymerase II transcription factor binding, was observed. This suggests an acute activation of gene expression critical for initiating adaptive responses and ensuring survival under stress. Concomitantly, photosynthesis-associated processes, including photosystem II, photosynthetic membrane, and chloroplast functions, were significantly downregulated during dehydration, indicating a shutdown of energy processes under desiccation conditions. Remarkably, these processes demonstrated partial recovery during the R2 phase, highlighting the resilience and adaptive recovery capabilities of the *S. caninervis* (Fig. 2E). Protein phosphorylation pathways also showed substantial modulation, suggesting key roles in altering protein function and signaling pathways essential for stress adaptation and recovery. Furthermore, nitrogen compound metabolism was actively regulated during both D2 and R1, emphasizing the plant's strategic management of nitrogen resources. Changes in lipid transport processes were particularly pronounced during dehydration, reflecting adjustments in membrane composition and the synthesis of protective compounds to mitigate water loss. Additionally, oxidoreductase activity was significantly enhanced, underscoring its role in maintaining redox homeostasis and managing oxidative stress during these challenging phases (Fig. 2E). From the KEGG pathway analysis, the degradation pathways of valine, leucine, and isoleucine were highly active during dehydration, highlighting the catabolism of branched-chain amino acids as a crucial adaptive strategy for energy generation and osmotic balance. Glutathione metabolism remained active throughout both D-R process, playing a pivotal role in detoxifying reactive oxygen species and protecting cellular integrity. During rehydration, there was a marked activation of ribosome biogenesis and protein processing in the endoplasmic reticulum, crucial for resuming normal protein synthesis and cellular functions. The synthesis pathways for cutin, suberine, and wax, as well as carotenoid biosynthesis, were also notably active, contributing to cellular protection and antioxidant defense during dehydration (Fig. 2F). This comprehensive molecular analysis elucidates the complex biological processes and pathways *S. caninervis* employs to navigate the challenges of D-R process. These findings highlight the *S. caninervis* robust adaptive strategies, which include transcriptional regulation, metabolic adjustments, and the restoration of cellular and metabolic functions.

### Proteome profiles of the dehydration and rehydration

**Fig. 3:**
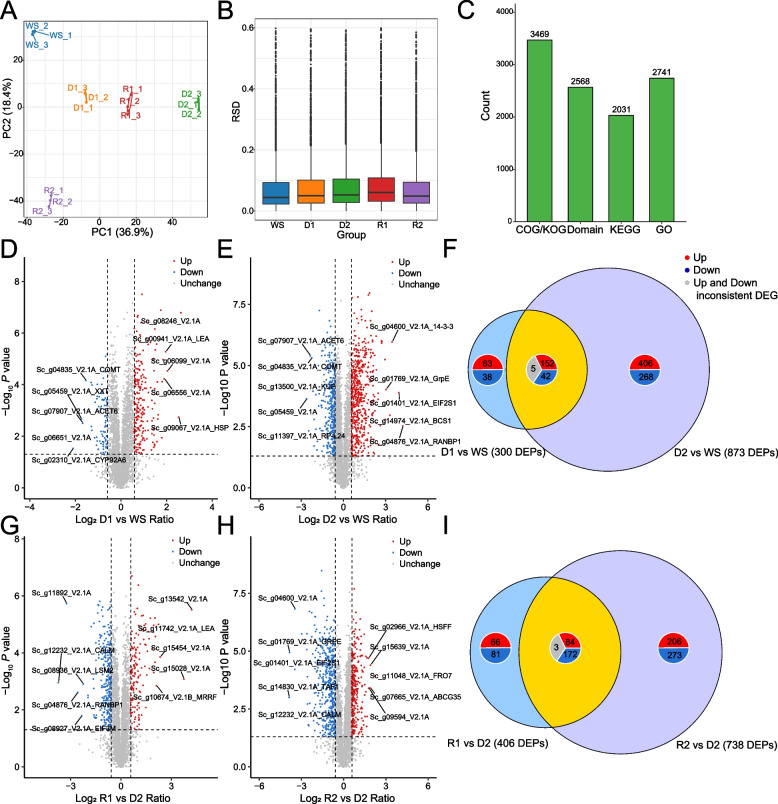
Proteome analysis of *S. caninervis* during D-R process. **A**. PCA of proteomics data. **B**. RSD of protein abundance across different treatment groups. **D**-**F**. DEPs analysis and Venn analysis during the dehydration stage. **G**-**I**. DEPs analysis and Venn analysis during the rehydration stage

The proteome analysis provides crucial insights into the molecular adaptations of *S. caninervis* to the D-R process typical in desiccation environments. This PCA plot depicts the distinct proteomic profiles among different sample groups, the variability captured by PC1 (36.9%) and PC2 (16.4%) underscores significant shifts in the proteome due to the D-R process responses (Fig. 3A). This relative standard deviation (RSD) demonstrates the variability within proteomic data groups, indicating tight reproducibility within some groups and higher variability in others, which reflect differential protein expression stability across different stress conditions (Fig. 3B). The COG/KOG domain category boasts the highest number of identified proteins, with a total count of 3,469. The KEGG classification encompasses 2,741 proteins, while the GO category accounts for 2,031 proteins (Fig. 3C) (Supplementary Material 1: Table S4). Examination of differentially expressed proteins (DEPs) during D1 vs WS revealed substantial changes in the proteome. A total of 300 DEPs were identified, with 215 upregulated and 85 downregulated proteins. Notably, several stress-responsive proteins, such as heat shock proteins, were markedly upregulated (Fig. 3D). As D2 vs WS, the number of DEPs increased dramatically to 873, with 563 upregulated and 310 downregulated proteins (Fig. 3E, F). This substantial increase in DEPs suggests a more pronounced reprogramming of the proteome as the severity of dehydration stress intensified. The overlap of 199 DEPs between D1 and D2 timepoints indicates a core set of proteins consistently modulated throughout the dehydration process. During the R1 vs D2, 406 DEPs were identified, with 153 upregulated and 81 downregulated proteins (Fig. 3G, I). This shift in protein expression reflects the initiation of recovery processes and the reactivation of metabolic functions suppressed during dehydration. This shift in protein expression reflects the initiation of recovery processes and the reactivation of metabolic functions suppressed during dehydration. Interestingly, several proteins involved in protein synthesis and energy metabolism showed upregulation, suggesting a rapid resumption of these essential cellular processes upon rehydration. The R2 vs D2 exhibited the most substantial proteome reorganization, with 738 DEPs identified (290 upregulated, 448 downregulated) (Fig. 3H, I). Notably, Many photosynthesis-related proteins showed significant upregulation during this phase, indicating a robust recovery of the photosynthetic. The overlap of 259 DEPs between R1 and R2 timepoints suggests a continuum of protein expression changes throughout the rehydration process. However, the larger number of unique DEPs at R2 compared to R1 indicates that the later stages of rehydration involve more extensive proteome remodeling, related to the full restoration of cellular functions and repair of desiccation-induced damage. In summary, the proteomic analysis reveals a dynamic and highly coordinated response of D-R process. The data highlight the plasticity of the proteome, demonstrating its ability to rapidly modulate protein expression to cope with extreme water loss and to efficiently recover upon rehydration.

**Fig. 4:**
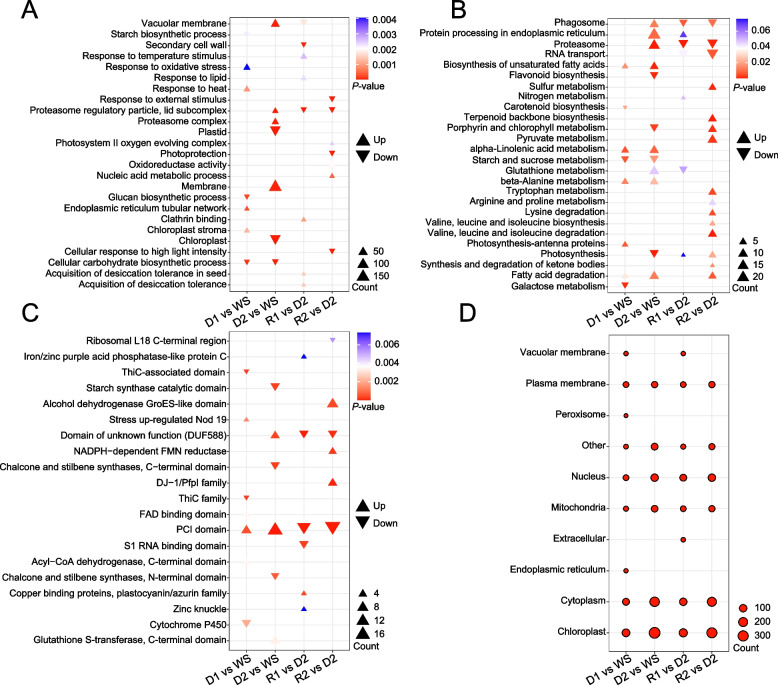
Enrichment analysis of DEPs in *S. caninervis* during the D-R process. **A**. GO functional enrichment of DEPs. KEGG functional enrichment of DEPs. **C**. Distribution of protein domains among DEPs. **D**. Predicted subcellular localization of DEPs. The size of the dots indicates the number of DEPs, and the color indicates the *p*-value

GO enrichment analysis of DEPs during the D-R process. A significant upregulation in genes involved in stress-related processes such as response to heat, oxidative stress, and temperature stimulus was observed, especially during D1 and R2. This indicates a robust activation of protective mechanisms against abiotic stress (Fig. 4A). In contrast, processes related to photosynthesis and chloroplast integrity were downregulated during D2, underscoring a metabolic shift towards conservation of energy and resources under stress conditions. There is a notable downregulation in genes related to photosystem II, chloroplast stroma, and light harvesting in photosynthesis. These processes are particularly reduced during the D2, reflecting a strategic reduction in energy metabolic activities to conserve resources in response to prolonged water deficit (Fig. 4A). The proteins involved in protein processing in the endoplasmic reticulum and proteasome complex exhibit varied expression patterns, with an initial decrease followed by a later increase during rehydration (Fig. 4A). This suggests a recalibration of protein synthesis and degradation mechanisms to adapt to and recover from dehydration stress. Upregulation of proteins involved in vacuolar membrane and endoplasmic reticulum tubular network indicates modifications in cell structure and compartmentalization, which are aimed at enhancing cellular resilience and maintaining homeostasis during dehydration (Fig. 4A). Significant upregulation in pathways such as starch and sucrose metabolism and arginine and proline metabolism are observed, particularly during the D1. These pathways are crucial for the synthesis of compatible solutes that act as osmoprotectants, helping the cells retain water and maintain turgor pressure, which is essential under water deficit conditions (Fig. 4B). The downregulation of photosynthesis-phenomena during dehydration indicates a strategic reduction in photosynthetic activity. This reduction helps conserve energy and reduce water loss through transpiration, demonstrating an adaptive response to minimize metabolic expenditure (Fig. 4B). Upregulation in biosynthesis of unsaturated fatty acids and nitrogen metabolism points towards modifications in lipid compositions and nitrogen utilization. These changes are aimed at enhancing membrane fluidity and efficiency in nitrogen use. Upregulation in biosynthesis of unsaturated fatty acids and nitrogen metabolism points towards modifications in lipid compositions and nitrogen utilization. Pathways such as glutathione metabolism and beta-alanine metabolism exhibit increased activity, particularly during rehydration phases. These pathways are integral to detoxifying reactive oxygen species (ROS) and other harmful byproducts of stress, thereby protecting cellular components from oxidative damage and aiding in the recovery process (Fig. 4B). Domains related to stress response like the heat shock proteins and alcohol dehydrogenase GroES-like domain showed an increase, particularly in D1, highlighting their roles in immediate stress mitigation (Fig. 4C). Thioredoxin (ThiC)-associated domain in maintaining redox homeostasis and promoting recovery from oxidative damage and Glutathione S-transferase, C-terminal structural domain also showed significant up-regulation, especially in R1. The subcellular localization of DEPs, revealing that the most significant changes occurred in the cytoplasm, chloroplast, and mitochondria. This distribution suggests a widespread cellular reprogramming to adjust metabolic and energy processes in response to dehydration. Notably, the increase in mitochondrial and chloroplast proteins during rehydration indicates a recovery phase, where energy production and photosynthetic activities are resumed (Fig. 4C).

### Variation in temporal dynamics of transcript and protein levels

Our integrative analysis of transcriptomic and proteomic data revealed distinct temporal dynamics and limited correlation between transcript and protein levels during the D-R process in *S. caninervis* (Supplementary Material 1: Fig. S3 A and B). We delved into the correlation between mRNA and protein expression during the D-R process of *S. caninervis*. During the dehydration stage, the difference between mRNAs and proteins was very obvious, and the Venn diagram showed that only 118 DEGs and DEPs overlapped (Supplementary Material 1: Table S5), and it was further found that the changes in mRNA and protein levels presented a very low correlation during the D-R process (Supplementary Material 1: Fig. S3 A and B). In the rehydration phase, a similar pattern of low correlation is observed, with only 22 DEGs and DEPs overlapping (Supplementary Material 1: Fig. S3 C and D).

**Fig. 5:**
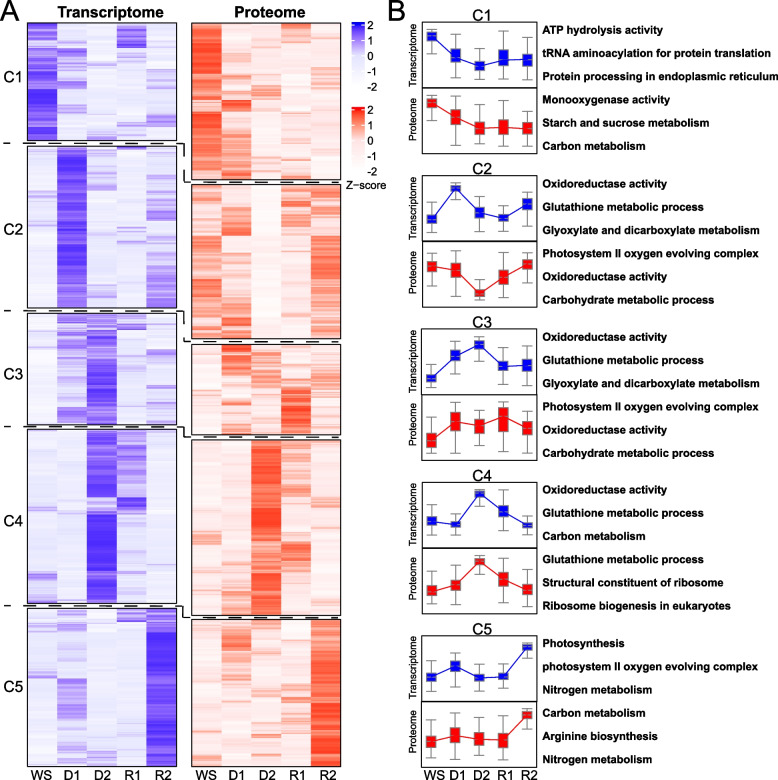
Integrative analysis of transcriptome and proteome of *S. caninervis* during the D-R process. **A**. Clustered heatmaps showing the expression patterns of transcripts (left) and their corresponding proteins (right) across five time points. Five distinct clusters (C1-C5) were identified based on expression patterns. Color scale represents z-score normalized expression values. **B**. Functional enrichment of clusters (C1-C5) in transcriptome and proteome. Clustering was performed using the Mfuzz algorithm implemented in the ClusterGVis R package. Transcript and protein abundance data were z-score normalized for standardization

Figure 5A presents clustered heatmaps of transcriptome and proteome data across five time points: WS, D1, D2, R1, and R2. The transcriptome heatmap shows five distinct clusters (C1-C5) with varying expression patterns. The proteome heatmap displays corresponding protein abundance changes, revealing both similarities and differences compared to the transcriptome patterns. Quantitative analysis of the clusters (Fig. 5B) (Supplementary Material 1: Table S6) revealed complex and divergent patterns between transcript and protein levels across the D-R process in *S. caninervis*. Cluster C1, associated with ATP hydrolysis, protein translation, and processing, as well as various metabolic processes, showed a general decrease in transcript levels throughout the D-R process, while corresponding protein levels initially increased slightly before declining. In C2, enriched for oxidoreductase activity and photosystem II functions, transcripts peaked at D1, contrasting with protein levels that were lowest at D2 and increased during rehydration. C3, sharing similar functional enrichments with C2, demonstrated elevated transcript levels during D2 and R1, while protein abundance remained relatively stable with a slight increase at R1. C4, involved in oxidoreductase activity, glutathione metabolism, and ribosome-related processes, exhibited highest transcript levels at D2 and R1, contrasting with protein levels that peaked at D2 but decreased during rehydration. C5, predominantly associated with photosynthesis, nitrogen metabolism, and arginine biosynthesis, displayed increased transcript levels at R2, whereas protein abundance gradually increased throughout the D-R process, peaking at R2. These results highlight the complex relationship between transcript and protein levels during the D-R process in *S. caninervis*, with significant variations in temporal dynamics across different functional categories. The observed discrepancies in expression patterns between transcripts and proteins within functionally related clusters underscore the importance of post-transcriptional regulation in modulating the plant's response to desiccation stress and subsequent recovery.

### Metabolite abundance dynamics during dehydration and rehydration

**Fig. 6:**
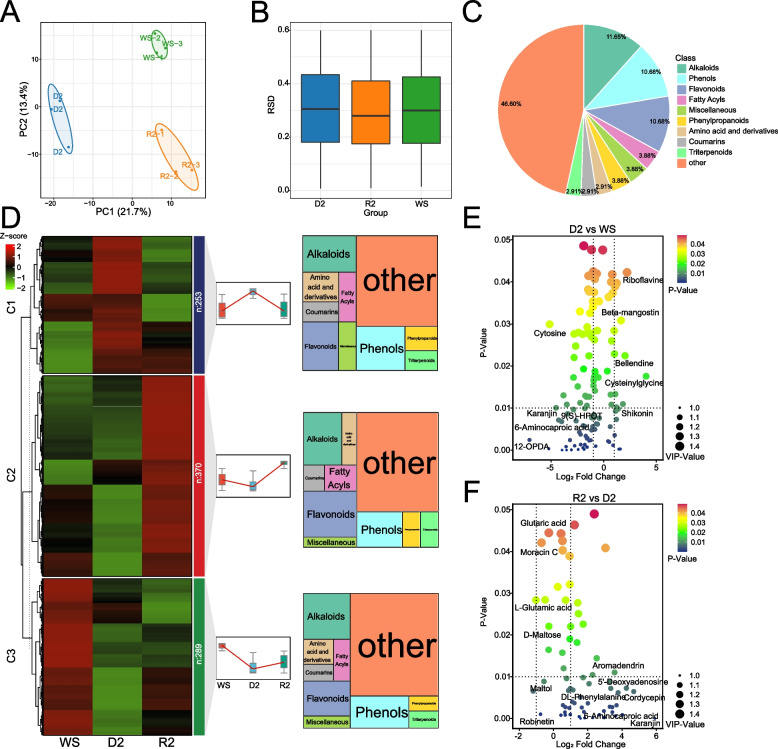
Metabolomic profiling of *S. caninervis* during D-R process. **A**. PCA of metabolomics data. **B**. RSD of metabolite abundance. **C**. Distribution of detected metabolites across Major chemical classes. Percentages indicate the proportion of each class among the 912 identified metabolites. **D**. Hierarchically clustered heatmap of z-score normalized metabolite abundances. Color scale represents z-score values. **E**, **F**. DAMs analysis. The size of the dot represents the VIP-value, and the color represents the *p*-value

Our comprehensive metabolomic profiling of *S. caninervis*, utilizing Liquid chromatography-mass spectrometry, unveiled an intricate landscape of 912 metabolites spanning 93 distinct classes. PCA of samples from each treatment group revealed significant metabolic shifts throughout the D-R process, with PC1 accounting for 21.7% of the total variance and clearly discriminating between extreme dehydration states (D2) and fully rehydrated samples (R2) (Fig. 6A). The RSD of protein abundance in *S. caninervis* under three distinct experimental conditions WS, D2, and R2 states (Fig. 6B). This visualization elucidates the variability in protein expression across diverse hydration conditions. While the median RSD values, denoted by horizontal Lines within each box, exhibit relative consistency at approximately 0.3 across all three conditions, notable disparities emerge in the distribution and spread of RSD values among the groups. The metabolome exhibited a rich profile dominated by alkaloids (11.65%), phenols (10.68%), and flavonoids (10.68%), followed by fatty acyls and phenylpropanoids (3.88%), and amino acids and derivatives, coumarins, and triterpenoids (2.91%) (Fig. 6C). Notably, several metabolites, including L-arginine, D-alpha-aminobutyric acid, p-octopamine, and various sugars (D-maltose, turanose, lactulose, and sucrose), maintained consistently high abundance throughout the D-R process (Supplementary Material 1: Table S7). To further elucidate the metabolic reprogramming, we employed orthogonal partial least squares discriminant analysis (OPLS-DA), which enabled the identification of 185 differentially abundant metabolites (DAMs), representing 20% of all detected metabolites, based on stringent criteria (variable importance in the projection [VIP] > 1 and *P* < 0.05) (Supplementary Material 1: Table S8).

The heatmap displays z-score normalized abundances of metabolites across three key physiological states: WS, D2, and R2 (Fig. 6D). The metabolites are hierarchically clustered into three main groups (C1, C2, and C3), each exhibiting distinct abundance patterns. C1 (*n* = 253) is characterized by metabolites that show the highest abundance during D2, with levels remaining elevated or partially increasing upon R2 compared to WS. This cluster is predominantly composed of alkaloids, flavonoids, and phenols, suggesting an important role for these compounds in desiccation tolerance and recovery. The abundance trend implies these metabolites serve protective functions during extreme water stress and contribute to maintaining cellular activities upon rehydration. C2 (*n* = 370) exhibits a pattern where metabolite abundances are highest in the rehydrated state (R2), with lower levels in both WS and D2 conditions. This cluster is rich in alkaloids, flavonoids, and phenols, but also includes a significant proportion of amino acids and derivatives, fatty acyls, and coumarins. The increased abundance during rehydration suggests these metabolites play crucial roles in recovery processes and the restoration of normal cellular functions. C3 (*n* = 289) displays a unique profile where metabolite abundances are highest in the water-saturated state (WS), decreasing during D2, and showing partial recovery upon R2. This cluster is primarily composed of alkaloids, flavonoids, and phenols, similar to the other clusters, but also includes amino acids and derivatives. The pattern suggests these metabolites may be important for normal cellular functions under hydrated conditions and are differentially regulated during the dehydration-rehydration cycle. Notably, alkaloids, flavonoids, and phenols are consistently prominent across all clusters, underscoring their multifaceted roles in the D-R process. The presence of amino acids and derivatives, particularly in C2 and C3, highlights the importance of nitrogen metabolism and protein turnover during water stress. In D2 vs WS (Fig. 6E), we observe a substantial metabolic shift as the moss transitions from a WS to D2. Notably, several metabolites show significant changes in abundance. Riboflavine and beta-mangostin exhibit marked increases (log_2_ fold change > 2, *p* < 0.05), suggesting a potential role in stress protection mechanisms. Bellendine and cysteinylglycine also show upregulation, which indicate increased antioxidant activity and altered amino acid metabolism under desiccation stress. Conversely, cytosine, karanjin, and 6-aminocaproic acid display significant downregulation. 12-OPDA (12-oxo-phytodienoic acid) shows a notable decrease, which imply alterations in jasmonic acid biosynthesis and signaling pathways under drought stress. In R2 vs D2 (Fig. 6F), Aromadendrin, 5'-deoxyadenosine, and cordycepin show substantial increases, indicating the activation of specific flavonoid biosynthesis and nucleotide salvage pathways during recovery. Karanjin and 6-aminocaproic acid exhibit significant upregulation, contrasting with their downregulation during dehydration. This suggests a rapid reversal of certain metabolic processes upon rehydration. Interestingly, some amino acids like L-glutamic acid and DL-phenylalanine show relatively modest changes, suggesting a controlled restoration of amino acid metabolism during early rehydration.

These metabolomic changes highlight the complex and dynamic nature of *S. caninervis* response to desiccation. The differential regulation of various metabolites, including flavonoids, amino acids, and nucleotide derivatives, underscores the multifaceted metabolic strategies employed by this moss species to withstand extreme water stress and rapidly recover upon rehydration. *S. caninervis* thus accumulates antioxidants to scavenge reactive oxygen species, accumulating nitrogenous amino acids and cytoprotective metabolites and decreasing energy metabolism to enter a protective state from dehydration-induced damage. During subsequent rehydration, many metabolites rapidly accumulated to prevent oxidative stress and restore physiological activities while repairing cells. This metabolic plasticity likely contributes significantly to the remarkable DT of *S. caninervis* in its arid desert habitat.

## Discussion

Extreme environments, such as arid and semi-arid regions covering approximately 41% of Earth's land surface, represent some of the most challenging habitats on our planet (Pravalie et al. 2016; Huang et al. 2017). These areas serve as natural laboratories for studying the limits of life and the remarkable adaptations of extremophiles. Within these harsh landscapes, biocrusts emerge as a testament to life's resilience, often forming the dominant biological cover and acting as critical ecosystem engineers. These complex communities play pivotal roles in stabilizing soils, conserving water, and fostering biodiversity in conditions where most life struggles to persist (Elbert et al. 2012; Rodriguez-Caballero et al. 2018). The global impact of these extreme communities is profound: biocrusts contribute an estimated 7% of Earth's annual terrestrial carbon uptake and account for nearly half of the biological nitrogen fixation by land-based organisms (Elbert et al. 2012). Among biocrusts, moss-dominated crusts represent the pinnacle of adaptation and complexity in these extreme habitats. As the most advanced form of biocrusts, moss-based communities, exemplified by species like *S. caninervis*, showcase extraordinary resilience to desiccation, temperature extremes, and high UV radiation (Stark et al. 2009; Li et al. 2024). Their ability to thrive in such hostile conditions not only significantly enhances desert ecosystem stability but also offers invaluable insights into the mechanisms of extreme stress tolerance. Understanding these adaptations is increasingly crucial as climate change expands arid zones globally, with implications ranging from ecological conservation to potential applications in agriculture and even astrobiology.

Our comprehensive multi-omics analysis of *S. caninervis* provides unprecedented insights into the molecular mechanisms underlying its exceptional desiccation tolerance. By integrating transcriptomic, proteomic, and metabolomic data, we have uncovered a complex and highly coordinated response to extreme water stress, revealing novel aspects of plant adaptation to arid environments.

### A phased recovery strategy in *S. caninervis*

Upon rehydration, Syntrichia caninervis exhibits a striking ability to rapidly restore its RWC and *Fv/Fm* within 12 to 24 h, while MDA levels remain elevated over the same period. This differential recovery underscores distinct timelines for physiological and cellular repair processes in this desiccation-tolerant moss. The swift restoration of RWC reflects the plant’s structural adaptations, such as efficient water-conducting tissues and leaf morphology, which prioritize rapid water uptake to reestablish cellular hydration-a critical survival mechanism. Similarly, the quick recovery of *Fv/Fm* indicates the prompt reactivation of photosynthetic machinery, enabling the moss to resume energy production and metabolic activity. In contrast, the persistently high MDA levels, a marker of lipid peroxidation caused by ROS during dehydration stress, suggest that oxidative damage is resolved more slowly. This prolonged recovery likely stems from the time-intensive activation of antioxidant systems (e.g., superoxide dismutase, catalase) and the repair or replacement of damaged cellular components, such as oxidized lipids. These findings highlight a phased recovery strategy in *S. caninervis*: rapid restoration of hydration and photosynthetic capacity as immediate survival priorities, followed by gradual mitigation of oxidative stress and cellular damage. Such a mechanism enhances the plant’s resilience to repeated dehydration-rehydration cycles, a trait shared with other desiccation-tolerant species (Costa et al. 2017; Farrant et al. 2015; Oliver et al. 2020; Yang et al. 2023b).

### Rapid and dynamic transcriptional reprogramming

The transcriptome analysis revealed extensive gene expression changes during the D-R process, with 3,153 DEGs identified at the point of complete dehydration. This massive transcriptional shift underscores the plant's ability to rapidly adapt its genetic program in response to extreme water stress. The scale of this response, involving approximately 25% of the total transcriptome, is notably larger than that observed in many other desiccation-tolerant plants, such as *Xerophyta viscosa* (Costa et al. 2017) or *Boea hygrometrica* (Zhang et al. 2013), suggesting a more comprehensive and intricate adaptation mechanism in *S. caninervis*. Genomic and transcriptomic analyses of the desiccation-tolerant grass *Sporobolus stapfianus* and its desiccation-sensitive sister species *Sporobolus pyramidalis* (Oliver et al., 2022) reveal distinct molecular responses to dehydration. While *S. pyramidalis* exhibits pronounced early transcriptome remodeling in response to water loss, *S. stapfianus* demonstrates a delayed peak in remodeling, occurring during severe dehydration, suggestive of transcriptional priming for desiccation tolerance. In contrast, *S. caninervis* displays a rapid and comprehensive multi-omics response during early dehydration, characterized by extensive transcriptional reprogramming, proteome remodeling (873 DEPs), and significant metabolite accumulation. These observations suggest that *S. caninervis* exhibits an enhanced preparedness for desiccation, likely an adaptive response to the extreme aridity of its native habitat, the Gurbantunggut Desert.

The machine learning-based network analysis highlighted the crucial roles of specific transcription factor families, particularly AP2/ERF, WRKY, bHLH, and HSF, in orchestrating this response (Supplementary Material 1: Fig. S2) (Chaffai et al. 2024; Ma et al. 2024; Song et al. 2023). The prominence of these factors aligns with findings in other stress-tolerant plants but appears more pronounced in *S. caninervis*, suggesting a more robust and finely tuned regulatory network. For instance, the AP2/ERF family, known for its involvement in abiotic stress responses (Nie et al. 2023; Ma et al. 2024), showed exceptionally high expression, indicating its pivotal role in extreme desiccation tolerance. Similarly, the enhanced expression of WRKY transcription factors, particularly WRKY12, suggests a unique regulatory mechanism in *S. caninervis*, as these factors are typically associated with both biotic and abiotic stress responses (Goyal et al. 2023).

The sustained expression of stress-responsive transcription factors during the rehydration process is particularly intriguing. This pattern suggests a form of “transcriptional memory,” potentially allowing *S. caninervis* to maintain a primed state for rapid response to subsequent desiccation events (Ding et al. 2012; Zuo et al. 2023). Such a mechanism could be crucial for survival in an environment characterized by unpredictable and limited water availability. This concept of transcriptional memory aligns with recent findings in other plants (Kapple et al. 2023) but appears to be more pronounced in *S. caninervis*, possibly due to the extreme nature of its habitat. In *B. hygrometrica*, mild drought acclimation triggers persistent rapid desiccation tolerance lasting at least four weeks, driven by DNA methylation shifts-hypomethylation during dehydration and hypermethylation post-acclimation-that pre-induce memory genes tied to RNA splicing, nutrient transport, and carbohydrate metabolism (Sun et al. 2021). Similarly, transcriptomic data in *S. caninervis* reveal altered expression of genes linked to RNA processing and metabolic regulation, alongside persistent protective metabolites post-dehydration, hinting at molecular priming. These parallels suggest that *S. caninervis* may leverage comparable epigenetic strategies to bolster tolerance to repeated stress, meriting further research into its stress memory mechanisms.

### Strategic energy management and cellular protection

The multi-omics analysis of *S. caninervis* reveals a sophisticated and highly coordinated response to extreme desiccation. At the broadest level, this moss exhibits a remarkable ability to rapidly shut down energy-intensive processes during dehydration while simultaneously activating a suite of protective mechanisms. This strategic reallocation of resources aligns with the “molecular switching” hypothesis proposed for resurrection plants (Farrant et al. 2015; Gechev et al. 2012; Hoekstra et al. 2001), but the scale and speed of these changes in *S. caninervis* suggest a more advanced implementation. The coordinated downregulation of photosynthesis-related genes and proteins, coupled with the swift upregulation of stress-responsive pathways across transcriptomic, proteomic, and metabolomic levels, indicates an exceptional degree of molecular integration evolved to cope with the extreme and unpredictable conditions of its desert habitat.

A key feature of *S. caninervis* transcriptomic and proteomic response was the marked upregulation of stress-responsive proteins, particularly heat shock proteins (HSPs), during dehydration. HSPs are known to act as molecular chaperones, preventing protein aggregation and facilitating proper protein folding under stress conditions (Becker and Craig. 1994; Wehmeyer and Vierling. 2000; Oliver et al. 2020). The sustained upregulation of HSPs throughout the D-R process suggests their critical role in maintaining proteome integrity and functionality under extreme water stress. This finding aligns with observations in other desiccation-tolerant species like *Boea hygrometrica* indicating a conserved role for molecular chaperones in extreme stress tolerance (Zhang et al. 2013; Liu et al. 2019). However, the extent and duration of HSPs upregulation in *S. caninervis* may represent a more robust and sustained protective mechanism, contributing to its exceptional DT. Furthermore, the metabolomic profile of *S. caninervis* unveils a distinctive composition rich in alkaloids, flavonoids, and phenols throughout the D-R process (Yang et al. 2023b). The prominence of these compounds, known for their antioxidant properties, hints at a specialized protective strategy that may contribute to the plant's extreme desiccation tolerance and rapid recovery capabilities (Challabathula et al. 2018; Oliver et al. 2020).

Moreover, the coordinated regulation of nitrogen metabolism pathways throughout the D-R process, evident at both transcriptomic and proteomic levels, suggests a crucial role for efficient nitrogen utilization in extreme stress tolerance. This emphasis on nitrogen management may represent a key adaptation to the nutrient-poor desert environment, enabling *S. caninervis* to maintain cellular integrity and rapidly resume normal functions under severe resource constraints.

### Membrane remodeling and cell wall modification

The multi-omics analysis of *S. caninervis* reveals a sophisticated and dynamic structural remodeling process that underpins its exceptional desiccation tolerance. This moss exhibits a remarkable ability to modify its cellular architecture in response to extreme water loss (Gao et al. 2015). The coordinated changes in membrane composition and cell wall structure represent a critical adaptation to the frequent and rapid transitions between hydrated and desiccated states characteristic of its desert habitat. These structural modifications likely contribute significantly to *S. caninervis* ability to withstand severe desiccation events and rapidly recover upon rehydration, positioning it as an ideal model for studying plant survival in extreme environments.

The transcriptomic and proteomic data reveal significant upregulation of lipid transport processes and cell wall-modifying proteins during dehydration. Notably, the differential expression of genes involved in cutin, suberine, and wax biosynthesis pathways (Fig. 2F) suggests a sophisticated mechanism for modifying the plant's protective outer layers. The coordinated activation of fatty acid desaturation pathways (Fig. 4B) indicates a critical strategy for maintaining membrane fluidity under extreme water loss. These changes, occurring rapidly and to a greater extent than observed in other desiccation-tolerant species (Dinakar et al. 2013; Gao et al. 2015; Giarola et al. 2017; Xu et al. 2021), likely enable *S. caninervis* to maintain cellular integrity even under severe water stress and facilitate its swift recovery upon rehydration. We observed upregulation of proteins involved in cell wall loosening and restructuring during dehydration, such as expansions and xyloglucan endotransglucosylases. These changes likely facilitate cell wall folding and prevent mechanical damage during water loss (Chen et al. 2020; Moore et al. 2009). Additionally, we noted increased abundance of proteins involved in membrane repair and stabilization, including lipid transfer proteins and dehydrins. These proteins may play crucial roles in maintaining membrane integrity during extreme water loss and facilitating rapid membrane rehydration. The coordinated regulation of cell wall and membrane-associated proteins likely contributes to *S. caninervis* ability to withstand extreme dehydration and rapidly recover upon rehydration.

The study uncovers specific mechanisms that underpin *S. caninervis* structural resilience. Metabolomics data revealed rapid synthesis of unique lipid compounds (phosphorylcholine, arachidonic acid, and methyl linoleate) during rehydration, which may play dual roles in membrane stabilization and signaling. These metabolites, not prominently reported in other resurrection plants, hint at novel aspects of structural adaptation in extreme desiccation tolerance. Furthermore, the early upregulation of cell wall-modifying enzymes during dehydration suggests a preemptive approach to cell wall adjustment, potentially enhancing the plant's ability to withstand mechanical stress during water loss. The integration of these structural changes with other protective mechanisms, such as the accumulation of compatible solutes and antioxidants, indicates a holistic approach to desiccation tolerance in *S. caninervis*.

### Unique metabolic reconfiguration

A key feature of *S. caninervis* metabolic response was the significant accumulation of compatible solutes during dehydration. We observed increased abundance of sugars (e.g., D-maltose, turanose, lactulose, and sucrose) and amino acids (e.g., L-arginine) throughout the D-R process. These compounds likely serve dual roles as osmoprotectants and energy reserves, facilitating cellular protection during dehydration and rapid recovery upon rehydration (Oliver et al. 2011; Yobi et al. 2013; ElSayed et al. 2014; Vidovic et al. 2022). However, the sustained high levels of these compatible solutes even during rehydration in *S. caninervis* suggest a more persistent protective strategy, potentially contributing to its superior DT and rapid recovery capabilities.

*S. caninervis* demonstrates unique features that set it apart from other desiccation-tolerant species. A distinctive characteristic is the significant modulation of nitrogen metabolism throughout the D-R process, evident at both transcriptomic and proteomic levels (Oliver et al. 2020). This emphasis on nitrogen-related pathways, more pronounced than in many other resurrection plants, suggests a crucial role for efficient nitrogen management in extreme desiccation tolerance (Dinakar et al. 2013). Furthermore, the metabolomic profile reveals a high abundance and diverse range of secondary metabolites, particularly alkaloids, flavonoids, and phenols, throughout the D-R process (Moore et al. 2009). The prominence of these compounds hints at a specialized protective strategy that may contribute to the plant's superior stress tolerance and rapid recovery capabilities.

The study uncovers specific metabolic mechanisms that underpin *S. caninervis* remarkable resilience. The rapid accumulation of unique metabolites such as riboflavin and beta-mangostin during dehydration, and aromadendrin and 5'-deoxyadenosine during rehydration, points to novel protective and recovery strategies not prominently reported in other desiccation-tolerant plants. The temporal dynamics of these metabolite changes, as revealed by the cluster analysis, indicate a phased metabolic response that aligns with the progression of the D-R process (Yang et al. 2023a). This phased approach may allow *S. caninervis* to optimize resource allocation and energy expenditure throughout the stress response and recovery phases. Moreover, the integration of these metabolic changes with other adaptive mechanisms, such as structural remodeling and gene expression regulation, suggests a holistic approach to desiccation tolerance in *S. caninervis*. This comprehensive strategy may explain the plant's superior ability.

### Temporal asynchrony between molecular levels

The integrative analysis of transcriptomic and proteomic data in *S. caninervis* reveals a striking temporal asynchrony between transcript and protein levels during the D-R process. This phenomenon, more pronounced in *S. caninervis* than in many other studied plants, highlights the complexity of post-transcriptional regulation in extreme desiccation tolerance responses (Xu et al. 2021; Dinakare et al. 2013). The Venn diagram analysis (Fig. S2) Starkly illustrates this disconnect, with only 118 overlapping DEGs and DEPs during dehydration, and a mere 22 during rehydration. Such a low degree of overlap is unusual compared to other stress-tolerant plants, suggesting a highly sophisticated and possibly unique regulatory mechanism at play in *S. caninervis* (Xu et al. 2021). This temporal disconnect between transcriptome and proteome changes may allow for more nuanced and flexible responses to rapid environmental fluctuations, potentially conferring a significant adaptive advantage in environments with extreme and unpredictable water availability (Moore et al. 2009; Xu et al. 2021). The clustered heatmaps (Fig. 5A) provide insights into the nature of this temporal asynchrony. The five distinct clusters (C1-C5) reveal complex and divergent patterns between transcript and protein levels across the D-R process. For instance, cluster C2, enriched for oxidoreductase activity and photosystem II functions, shows peak transcript levels at D1, contrasting sharply with protein levels that are lowest at D2 and increase during rehydration. This pattern differs from observations in other desiccation-tolerant plants, where photosynthesis-related transcripts and proteins often show more synchronized downregulation during desiccation (Costa et al. 2017; Farrant et al. 2015; Xu et al. 2021). Interestingly, the asynchrony is not uniform across all functional categories. Cluster C5, predominantly associated with photosynthesis, nitrogen metabolism, and arginine biosynthesis, shows a more coordinated response, with both transcript and protein levels gradually increasing throughout the D-R process. This selective synchronization implies a prioritization of certain pathways crucial for recovery, a strategy that may contribute to *S. caninervis* rapid rehydration response.

These observations suggest sophisticated post-transcriptional regulatory mechanisms in *S. caninervis* that fine-tune its cellular state in response to changing water availability. Such mechanisms might include differential mRNA stability, selective translation, or protein turnover regulation, which could be more advanced in *S. caninervis* compared to other resurrection plants. The maintenance of elevated transcript levels for certain genes, even when corresponding protein levels have decreased, could represent a form of molecular memory that allows *S. caninervis* to respond more rapidly to subsequent desiccation events (Bruce et al. 2007). Future research focusing on the specific mechanisms underlying this temporal decoupling, such as the roles of RNA-binding proteins, non-coding RNAs, or post-translational modifications, could provide valuable insights into enhancing stress tolerance in crops and developing strategies for plant survival in extreme environments.

### Future directions

**Fig. 7:**
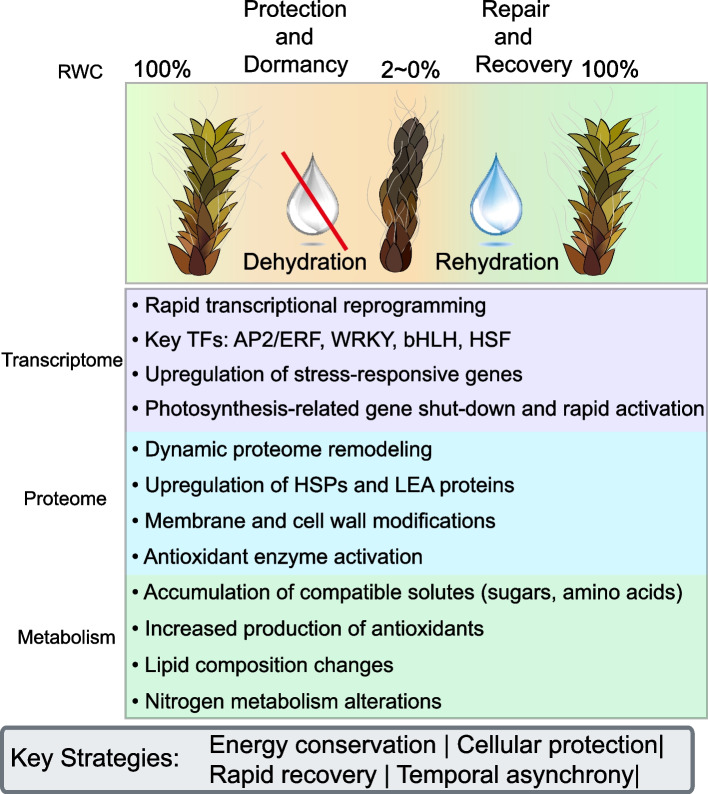
Summary of major events and key adaptation strategies that occur during the D-R process in *S. caninervis*

Our comprehensive multi-omics analysis of *S. caninervis* provides a holistic view of the molecular strategies employed by this remarkable plant to survive extreme desiccation. The integration of transcriptomic, proteomic, and metabolomic data has unveiled a complex interplay between different levels of cellular regulation that collectively contribute to extreme stress tolerance (Fig. 7). These findings not only advance our fundamental understanding of plant adaptation to water stress but also open new avenues for addressing pressing challenges in agriculture and beyond.

The unique adaptations observed in *S. caninervis*, such as its sophisticated transcriptional regulation, advanced energy management strategies, and novel metabolic reconfigurations, offer promising targets for improving crop resilience to water stress. Future research should focus on elucidating the specific roles of the novel metabolites identified in this study and unraveling the complex post-transcriptional regulatory mechanisms that enable *S. caninervis* rapid and flexible stress responses.

Moreover, the extreme resilience of *S. caninervis*, particularly its ability to withstand conditions analogous to those on Mars, positions it as a valuable model for studying plant adaptation to extraterrestrial environments (Li et al. 2024; Pan and Lin. 2024). This opens up exciting possibilities for Astro biological research and the development of plants suitable for off-world cultivation.

## Conclusion

This comprehensive multi-omics analysis of *S. caninervis* reveals unprecedented insights into the molecular mechanisms underlying extreme desiccation tolerance in desert plants. Our findings demonstrate a sophisticated stress response system characterized. The observed temporal asynchrony between transcript and protein levels, coupled with the unique accumulation of protective metabolites and coordinated regulation of energy management pathways, suggests a highly evolved adaptation strategy that enables rapid transitions between dormancy and recovery. These findings not only advance our understanding of plant evolution in extreme environments but also provide valuable targets for enhancing crop resilience to water stress and potential applications in astrobiology. The remarkable adaptations of *S. caninervis* offer crucial insights for addressing agricultural challenges in an increasingly arid world and expand our understanding of the limits of plant life under extreme conditions.

## Methods and materials

### Plant materials, growth conditions and sampling

Dry samples of *Syntrichia caninervis* were collected from natural populations in the Gurbantunggut Desert, Xinjiang, Northwest China (Fukang County, 44°32′30″N, 88°6′42″E). The sampling site was selected due to its representative arid conditions and the dominance of *S. caninervis* in the biological soil crusts (BSCs). Entire gametophyte clusters, including both leafy shoots and basal rhizoids, were harvested from the upper 2–3 cm of the soil crust using sterile forceps and placed into airtight, sterile polyethylene bags to maintain their desiccated state. This tissue selection was based on the gametophyte being the primary photosynthetic and desiccation-tolerant structure of the moss, making it the most relevant for studying dehydration-rehydration (D-R) responses. Approximately 50–60 individual clusters (each ~ 1–2 cm^2^) were collected from three distinct 1 m^2^ plots, spaced at least 10 m apart, to account for spatial heterogeneity. These samples were pooled per plot to form three independent biological replicates, ensuring robust statistical representation of the population.

Upon return to the laboratory, samples were stored at 25 °C in darkness within sealed containers to preserve their dormant, desiccated state until experimental processing. For the D-R experiments, desiccated gametophytes were rehydrated by submerging them in ultrapure water for 24 h at 25 °C under a 16-h light/8-h dark photoperiod (50 µmol photons m⁻^2^ s⁻^1^) to achieve a fully water-saturated (WS) state, serving as the control condition. Subsequently, a slow-drying protocol was implemented by placing the rehydrated samples in a controlled environment at 25 °C and 66.67% relative humidity (−57 MPa) (Liu et al. 2021; Yang et al. 2023b). Samples were collected at two dehydration time points: 6 h (D1, early dehydration) and 48 h (D2, full dehydration). For rehydration, fully dehydrated samples (D2) were transferred to Petri dishes Lined with filter paper saturated with ultrapure water at 25 °C under the same Light conditions. Rehydrated samples were harvested at 0.5 h (R1, early rehydration) and 24 h (R2, full rehydration), with D2 serving as the reference control.

For each time point (WS, D1, D2, R1, R2), approximately 200 mg (fresh weight) of gametophyte tissue was sampled from each of the three biological replicates. Each replicate was derived from a distinct plot to ensure independence. Samples were immediately flash-frozen in liquid nitrogen post-harvest and stored at −80 °C until RNA, protein, and metabolite extraction. This sampling strategy was designed to capture the dynamic molecular responses across the D-R continuum while maintaining consistency in tissue type and experimental conditions.

### Physiological indicator measurements

RWC curves were produced using the formula RWC% = (*W*_f_ – *W*_d_)/(*W*_t_ – *W*_d_) × 100, where *W*_f_ indicates the fresh weight measured at every time point during the D-R process, *W*_d_ indicates the weight measured after drying for 48 h at 80 °C, and *W*_t_ indicates the saturated fresh weight of samples (Rathnayake et al. 2019).

Ptimal photochemical efficiency of photosystem II (*F*_v_*/F*_m_) was determined using a portable modulated fluorometer (PAM-2500; Heinz Walz, Germany). The saturation pulse method, with parameter settings recommended by Zhang et al. (2011), was employed to calculate *Fv/Fm* after dark adaptation by covering the sample for over 30 min.

The activities of the malondialdehyde (MDA) were measured using detection assay kits (Nanjing Jiancheng Bioengineering Institute, Nanjing, China), according to the manufacturer’s instructions.

### Library preparation and transcriptome assembly

mRNA was isolated from total RNA using poly-T oligo-attached Magnetic beads. First-strand cDNA synthesis was performed using random hexamers and M-MuLV Reverse Transcriptase, followed by second-strand synthesis with DNA Polymerase I and RNase H. After end-repair and 3'adenylation, adaptors were Ligated. Fragments of 370–420 bp were selected and PCR-amplified using Phusion High-Fidelity polymerase. The Library quality was assessed using an Agilent Bioanalyzer 2100. Only RNA samples with integrity numbers above 8 were used. Sequencing was performed on an Illumina Novaseq platform with 150 bp paired-end reads.

### RNA-Seq analysis and qRT-PCR validation

Raw fastq data were processed using fastp to obtain clean reads. High-quality data (Q20, Q30, and GC content) were used for downstream analyses. The reference genome and annotations were obtained from Gao et al. (2024). Hisat2 v2.0.5 was used for genome indexing and read alignment. Transcripts were assembled and quantified using StringTie v1.3.3b (Pertea et al. 2015). Gene expression levels were calculated as FPKM using FeatureCounts v1.5.0-p3. Differential expression analysis was performed using DESeq2 R package (v1.20.0). Genes with adjusted *p*-value ≤ 0.05 and |Log_2_ fold change|≥ 1 were considered differentially expressed. GO and KEGG pathway enrichment analyses were conducted using the clusterProfiler R package.

RT-qPCR validation was performed on selected DEGs using a CFX96 Real-Time PCR System (Bio-Rad). *ScTubulin* served as the reference gene, and relative abundance was calculated using the 2^−ΔΔCt^ method. Each experiment included three biological and technical replicates.

### Analysis of proteomes

Proteins were extracted from ground samples using TCA/acetone precipitation, followed by phenol extraction. For PTM studies, relevant inhibitors were included in the lysis buffer. Protein concentration was determined using a BCA kit. Proteins were precipitated with TCA, washed, and digested with trypsin overnight. Peptides were reduced, alkylated, and desalted using a Strata X SPE column. LC–MS/MS analysis was performed on a NanoElute UHPLC system coupled to a timsTOF Pro mass spectrometer (Bruker Daltonics) operated in PASEF mode. MS/MS data were processed using MaxQuant (v.1.6.15.0) against the *S. caninervis* database. Carbamidomethylation of cysteine was set as a fixed modification, with protein N-terminal acetylation and methionine oxidation as variable modifications. The false discovery rate was adjusted to < 1% for proteins, peptides, and PSMs.

Protein quantification was based on the mean relative quantification values across three replicates. Differentially abundant proteins were identified using t-tests on log_2_-transformed values (*p* < 0.05). Functional annotation was performed using eggnog-mapper for GO analysis, PfamScan for domain annotation, and KEGG for pathway analysis. Subcellular localization was predicted using PSORTb. Functional enrichment was assessed using Fisher's exact test (fold enrichment > 1.5, *p*-value < 0.05).

Transcriptome-proteome correlation analysis was conducted using the ggplot2 R package. Heatmaps were generated using the ClusterGVis R package, employing z-score normalization and the Mfuzz algorithm for clustering.

### Analysis of metabolomic

Freeze-dried samples were pulverized and extracted using a methanol:water (3:1, v/v) solution containing an internal standard. UHPLC-MS analysis was performed on an EXIONLC System (Sciex Technologies) with ACQUITY UPLC HSS T3 columns (Waters). The mobile phase consisted of 0.1% formic acid in water and acetonitrile, with a flow rate of 400 μL/min. A Sciex QTrap 6500 + mass spectrometer was used for detection.

Data acquisition utilized Multiple Reaction Monitoring (MRM) via SCIEX Analyst Work Station Software (v.1.6.3). Peak detection and annotation were performed using an in-house R program (v.4.2.2) with the KEGG pathway database.

Statistical analysis included OPLS-DA modeling using SIMCA software. Model validation was conducted through seven-fold cross-validation and permutation tests. Significant metabolites were identified based on *p*-value < 0.05 (Student's *t*-test), VIP > 1, and fold-change < 0.5 or > 2.

### Data visualization and statistical analysis

The visualizations in this article are all drawn using R (v.4.2.2). PCA analysis was plotted using the ggplot2 R package. Venn analysis was plotted using the GOplot R package. Heatmaps for cluster analysis were drawn using the ClusterGVis R package. To perform k-means clustering, the metabolite abundance values were first normalized using z-scores. The data were analyzed using a one-way analysis of variance (ANOVA) in SPSS 25 (IBM, Armonk, New York, USA). Significant differences among treatments were tested based on the least significant difference at *p* < 0.05. The bar graphs were generated using GraphPad Prism 9 software. Adobe Illustrator CC 2023 was used for image processing.

## Supplementary Information

Supplementary Material 1.

## Data Availability

The datasets mentioned in this study are available in a published article and various online repositories. The data supporting the findings of this study have been deposited into the CNGB Sequence Archive of the CNGB under the accession number CNP0003722 and CNP0006308 (https://db.cngb.org/).

## Abbreviations

DT: Desiccation Tolerance
D-R: Dehydration-Rehydration
RWC: Relative Water Content
*Fv/Fm*: Optimal Photochemical Efficiency of Photosystem II
MDA: Malondialdehyde
ROS: Reactive Oxygen Species
DEGs: Differentially Expressed Genes
DAMs: Differentially Abundant Metabolites
PCA: Principal Component Analysis
RSD: Relative Standard Deviation
DEPs: Differentially Expressed Proteins
OPLS-DA: Orthogonal Partial Least Squares Discriminant Analysis
